# Effective Method for Obtaining the Hydrosols of Detonation Nanodiamond with Particle Size < 4 nm

**DOI:** 10.3390/ma11081285

**Published:** 2018-07-25

**Authors:** Andrei D. Trofimuk, Diana V. Muravijova, Demid A. Kirilenko, Aleksandr V. Shvidchenko

**Affiliations:** 1 Ioffe Institute, 194021 St. Petersburg, Russia; trofimuk.ad@mail.ioffe.ru (A.D.T.); demid.kirilenko@mail.ioffe.ru (D.A.K.); 2 ITMO University, 197101 St. Petersburg, Russia; diana.muraveva.93@mail.ru

**Keywords:** detonation nanodiamond, colloids, size separation

## Abstract

Detonation nanodiamond is a commercially available synthetic diamond that is obtained from the carbon of explosives. It is known that the average particle size of detonation nanodiamond is 4–6 nm. However, it is possible to separate smaller particles. Here we suggest a new approach for the effective separation of detonation nanodiamond particles by centrifugation of a “hydrosol/glycerol” system. The method allows for the production of the detonation nanodiamond hydrosol with a very sharp distribution in size, where more than 85% of particles have a size ranging 1–4 nm. The result is supported by transmission electron microscopy, atomic force microscopy, and dynamic light scattering.

## 1. Introduction

Detonation nanodiamond (DND) is a commercially available carbon nanomaterial with particle sizes of 4–6 nm produced by detonation synthesis from the carbon of explosives that can be used for various applications, including bioapplications [1,2,3,4]. Reduction of diamond particles’ size would open perspectives for using of nanodiamonds as a basis for electronic quantum devices [5], stable photoemitters [6], and, probably, reflectors of cold neutrons [7]. Moreover, calculations show that diamond particles with a size of 1–3 nm have a non-uniform surface electrostatic potential [8], which, apparently, leads to the formation of gels at low concentrations of nanodiamond in water [9].

A wide range of applications causes high demands on the production of DND particles. It concerns not only the purification of nanodiamonds from detonation by-products [10], but also the production of individual DND particles with a narrow size distribution. The first problem was solved long time ago but the solution of the second problem was greatly hampered by the agglomeration of DND particles. But now several methods for obtaining hydrosols of individual DND particles are available [11,12,13,14]. It had allowed for the separation of individual particles by size [15].

The subsequent separation of nanoparticles by their size is usually carried out by centrifugation of sols, and now it is a standard practice (see, for example, References [15,16,17]). However, the method of simple centrifugation has a number of flaws. The main ones are the difficulty of obtaining narrow particle size distributions and a significant reduction in the concentration of particles (compared to the original concentration). As a rule, the most effective methods for the separation of particles are centrifugation methods using a density gradient [18].

The method proposed in this work allows for an increase in the efficiency of separation of individual particles by sizes with preservation of the solid phase yield. Furthermore, we suggest that our method can be used as the first steps to obtaining the smallest DND particles. The DND hydrosols obtained by our method have a narrow particle size distribution.

## 2. Materials and Methods

The commercial DND powder produced by Scientific and Production Closed Joint-Stock Company “Sinta” (Minsk, Republic of Belarus) was used in this work. We purified commercial DND from metal impurities by multiple treatments with a solution of hydrochloric acid under ultrasonic irradiation [10]. The nanodiamond was repeatedly washed with demineralized water to remove residual acid after such treatment.

Experiment was carried out for three types of hydrosols of individual DND particles: hydrosol prepared by method submitted in Reference [13] (named as DND-St), hydrosol after simple centrifugation (named as DND-SC), and hydrosol after modified centrifugation (named as DND-MC). The DND-SC and DND-MC hydrosols were obtained from DND-St hydrosol.

DND-St hydrosol was prepared as follows: (1) purified DND powder was annealed in air at 450 °C for 6 h; (2) the annealed powder was dispersed in demineralized water by ultrasonic treatment; and (3) the obtained hydrosol was centrifuged (relative centrifugal force RCF = 1.8 × 10^4^ g, revolutions per minute RPM = 1.4 × 10^4^, centrifugation time t = 40 min, time at RPM = 38.5 min, mean-effective centrifugation radius MECR = 5.9 cm), such that the supernatant retrieved after centrifugation was DND-St hydrosol.

The DND-SC hydrosol was prepared as follows: (1) the DND-St hydrosol (mass of hydrosol was 6 g, height of hydrosol column in capsule was 5.0 cm) was poured into capsules and centrifuged (RCF = 6 × 10^4^ g, RPM = 2.6 × 10^4^, t = 40 min, time at RPM = 37.5 min, MECR = 5.9 cm); (2) the top part of the supernatant with volume 2 mL was carefully removed after centrifugation. This part was the DND-SC hydrosol.

The DND-MC hydrosol differed from DND-SC in such a way that it was centrifuged using glycerol on the bottom of capsules. Sample DND-MC was prepared as follows: (1) glycerol (mass of glycerol was 3 g) was poured into capsules, then DND-St hydrosol (mass of hydrosol was 3 g, height of “hydrosol/glycerol” system column in the capsule was 4.4 cm) was carefully poured over glycerol, then the capsules were centrifuged (RCF = 6 × 10^4^ g, t = 40 min, time at RPM = 37.5 min, MECR = 6.1 cm); (2) the top part of supernatant with volume 2 mL was carefully removed from the capsules after centrifugation and purified from glycerol by dialysis. Dialysis of the hydrosol was carried out using Cellu-Sep dialysis bags with a pore size of 12–14 kDa. It should be specially noted, that dialysis was carried out for DND-MC hydrosol only.

Centrifugation of all investigated hydrosols was performed by centrifuge Sigma 3-30KS (rotor 12,158; Sigma Laborzentrifugen GmbH, Osterode am Harz, Germany) in standard medical polypropylene capsules (capsule height 7.5 cm, capsule volume 10 mL) using the modified adapters [19].

Analysis of the degree of contamination with glycerol was carried out by Fourier transform infra-red spectroscopy (FTIR) using the Infralum FT-08 spectrometer (Lumex GC, St. Petersburg, Russia) with a diffuse reflection accessory. The hydrosol was dried at 130 °C to a constant weight. Dried DND powder was mixed with KBr in a ratio of 1:4, respectively, before measurement.

The size distributions of DND particles in hydrosols were analyzed using dynamic light scattering (DLS) using an analyzer Zetasizer Nano ZS (Malvern Instruments, Worcestershire, UK).

Samples for particle size analysis were performed using transmission electron microscopy (TEM) were prepared by drying the DND hydrosol on the surface of a copper grid for electron microscopy with a hole diameter of 100 μm. A graphene membrane was formed in the grid holes by the method described in Reference [20]. Measurements were carried out using a microscope JEM-2100F (Jeol, Tokyo, Japan).

Particle size analysis using atomic force microscopy (AFM) was performed using a microscope Dimension 3100 (Veeco, New York, NY, USA). A cantilever with a needle radius of 2 nm was used (TESP-SS, Bruker, Hamburg, Germany). Nanodiamond was applied on the surface according to the technology of PELCO (Ted Pella, Inc., Redding, CA, USA). recommended by the manufacturer for similar nanoparticles of colloidal gold [21]. The substrate of mica was refreshed using the method of flaking the atomic layers by means of an adhesive tape to obtain an atomically smooth surface. Then the surface was treated with Poly-l-Lysine, where a drop of Poly-l-Lysine, 20 μL in volume, was applied for 60 s, then it was washed off with distilled water. The drop of the nanodiamond hydrosol had a volume of about 20 μL. The concentration of DND particles in hydrosol was 5 × 10^−4^ wt%. The drop was kept on the substrate for 10 min, and then it was pumped out with a pipette, after which the sample was dried at 40 °C for 5 min. Statistical data of the diamond nanoparticles sizes were obtained by analyzing topographic images with a size of 5 μm × 5 μm. The substrate imperfection and the insufficient resolution of closely located nanoparticles due to the finiteness of the rounding radius of the AFM probe required usage of a special topographic image processing technique that eliminated particle loss in image analysis [22].

## 3. Results and Discussion

**Figure 1:**
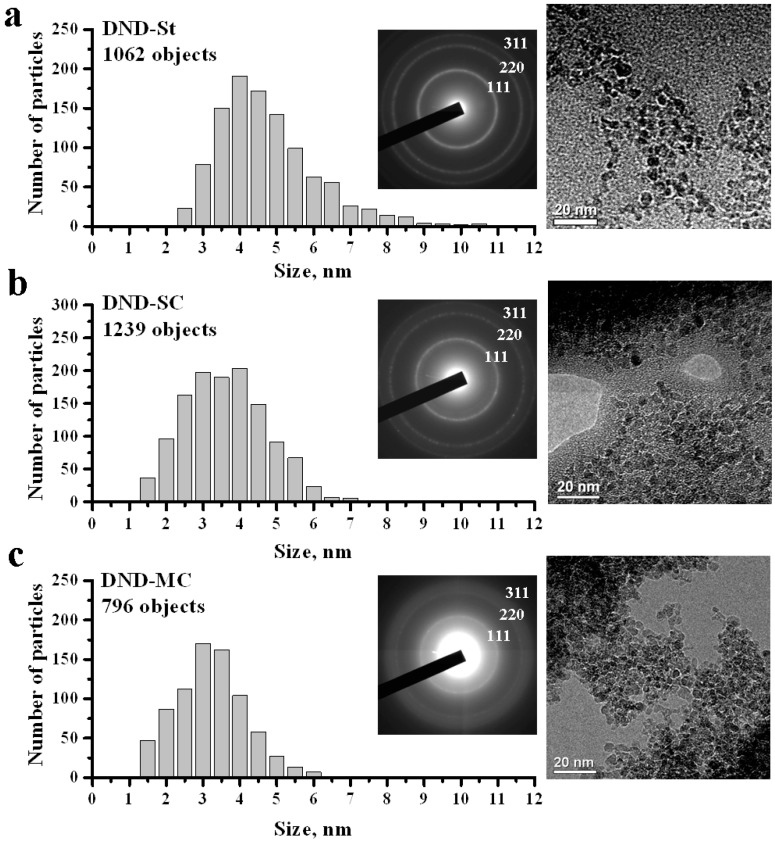
Particle size distributions for DND-St (**a**), DND-SC (**b**), and DND-MC (**c**) samples obtained using the TEM method, with their corresponding electron diffraction patterns and TEM images.

Firstly, we made sure that our method could not be cause physical or chemical changing of particles in any way. That was proved by method of electron diffraction. The electron diffraction patterns corresponding to explored samples, and particle size distributions based on TEM images are presented in Figure 1. The diffraction patterns were typical for polycrystalline samples of nanoscale diamond: the diffraction rings of reflections (111), (220), and (311) are clearly visible. Polycrystalline structures were formed in a process of hydrosol drying on a graphene membrane. It can be seen that the polycrystalline structures consisted of single-crystalline diamond particles (see Figure 1).

Secondly, we observed that the chosen method of hydrosol preparation influenced the change of particle size distribution and yield of the main type of particle. This observation was made using TEM, AFM, and DLS measurements.

Analysis of the TEM images of the DND-St sample confirmed that the sample consisted of nanometer-sized particles; the particle size distribution varied in the range from 2 nm to 11 nm, while the majority of particles had a size of about 4 nm (see Figure 1a). From the size distribution of the DND-SC sample (see Figure 1b), it can be seen that simple centrifugation resulted in a shift of distribution to the 2.5 nm to 4.5 nm region, and the distribution is noticeably broadened. The contribution of particles larger than 5 nm remained significant. Thus, simple centrifugation did not allow separation of particles 4 nm in size and smaller from larger particles. At the same time, the shift of the distribution maximum to a region from 2.5 nm to 3.5 nm was observed after centrifugation of the “hydrosol/glycerol” system (DND-MC sample), and the distribution became narrower (see Figure 1c). The smoother diffraction pattern observed for this sample is explained by the fact that both the average particle size and the fraction of large particles decreased. It is worth noting that the distributions constructed from the analysis of TEM images gave information about the sizes of single-crystal particles only. In view of the limited resolution of the microscope, there arose a question about the reliability of the measurement results in the region of less than 2 nm.

**Figure 2:**
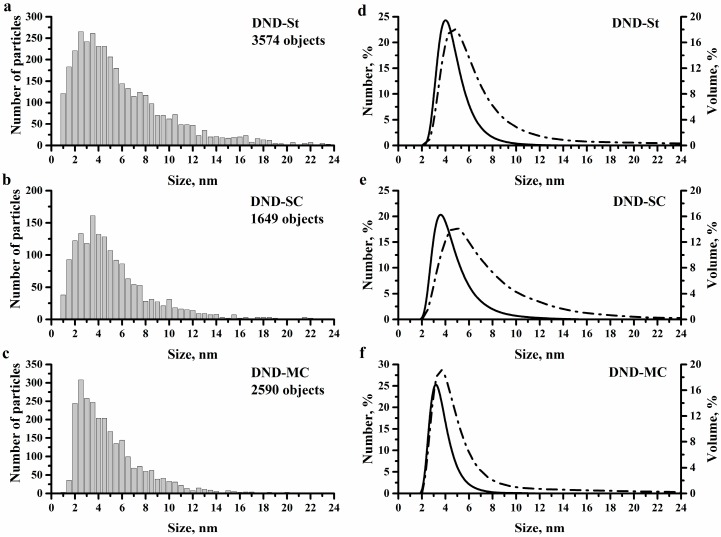
Particle size distributions obtained using the AFM (**a**–**c**) and DLS (**d**–**f**) methods for investigated samples. The DLS particle size distributions by number are represented by a solid line, while distributions by volume are represented by the dash dot lines.

The AFM method was additionally used to answer the question of whether the particles with sizes less than 2 nm were present. The size distributions of DND particles obtained by the AFM method are shown in Figure 2. It can be noted that the particles smaller than 2 nm were present in all samples, and this result can be considered statistically significant. However, their number decreased in the case of the DND-MC sample (Figure 2c). Some of those particles were possibly removed during the dialysis process. The distributions of the initial sample (DND-St) and the sample obtained by simple centrifugation (DND-SC) had no significant differences in their maximums. The result obtained after modified centrifugation (DND-MC) differed strongly from the previous two distributions. A shift of the maximum of the distribution to the region of 2–3 nm was observed. Moreover, the DND-MC sample had a narrow size distribution in comparison with the DND-St and DND-SC samples.

All of the AFM distributions were very different from the TEM results for sizes greater than 6 nm. This may have been due to agglomerates left after centrifugation of the hydrosol, and/or aggregates formed during the sample preparation for AFM analysis. The analysis of the DND particle sizes in hydrosols by the DLS method was carried out for better understanding why large particles appeared. The size distributions obtained using the DLS method are shown in Figure 2. Comparison of the size distributions obtained using AFM and DLS methods indicated that most of the large particles (>6 nm) are formed during the preparation of the samples for AFM analysis. This may have been due to the formation of aggregates from DND particles owing to the action of van der Waals forces in the process of the hydrosol drying on a substrate. It is also worth noting that the centrifugation resulted in a shift of the distribution maximum to a region of smaller sizes. In the case of the DND-MC sample, the maximum of the distribution was shifted to the region of 3.0–3.5 nm. As seen, the DLS method gave an overestimated value of the distribution maximum in comparison with the AFM and TEM methods, which can be explained by the fact that the hydrodynamic diameter of particles is the result of DLS measurements. Comparing the obtained results, it can be assumed that centrifugation of system “hydrosol/glycerol” (Figure 2f) made it possible to get a hydrosol of individual DND particles with a maximum of size distribution in the region of 3 nm and remove most of the particles larger than 8 nm from the sample in contrast to simple centrifugation.

Centrifugation of hydrosols inevitably led to a decrease in the concentration of particles in them. Therefore, one of the stages of analysis of the resulting supernatants was the determination of the yield of the solid phase. To this end, the mass measurements of DND particles in the obtained samples were made for the DND-SC and DND-MC hydrosols. The yield of the DND particles was 14% (for DND-SC) and 13% (for DND-MC) from the mass of particles in the initial hydrosol (DND-St hydrosol). Thus, the simple centrifugation and modified centrifugation resulted in close values of the final mass of DND particles in hydrosols.

**Figure 3:**
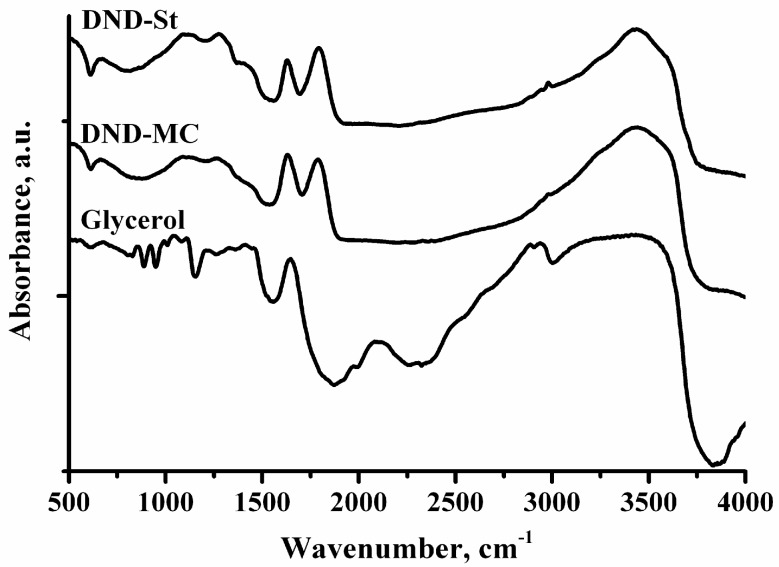
FTIR spectra of DND-St and DND-MC samples, and glycerol.

However, it should be noted that the method of centrifugation using glycerol led to inevitable contamination of the supernatant with glycerol. Therefore, one of the steps in obtaining a DND-MC sample is dialysis. Efficiency of dialysis for this purpose was proved using FTIR spectroscopy. The FTIR spectra of the DND-St and DND-MC samples are shown in Figure 3. The spectrum of the DND-SC sample was identical to the DND-St spectrum, which is why it is omitted. The spectrum of pure glycerol is also presented for comparison. It can be seen that the band corresponding to the vibrations of the carbonyl groups on the nanodiamond surface at 1760 cm^−1^ was manifested in both samples, and there are no characteristic bands of glycerol. Thus, it can be argued that dialysis was an effective method of purifying nanodiamonds from glycerol.

In summary, it can be noted that the centrifugation of system “hydrosol/glycerol” allowed for the production of DND hydrosols with a very sharp size distribution when more than 85% particles had sizes ranging from 1 nm to 4 nm (see DND-MC sample on Table 1). These results were confirmed using TEM and DLS methods. The distributions obtained using the AFM method gave the understated values for all samples. This was due to the aggregation of particles in the process of hydrosol drying on the substrate.

## 4. Conclusions

The use of the “hydrosol/glycerol” system in the centrifugation of DND hydrosols proved to be a fairly simple method of efficient particle size separation. This method allowed for obtaining DND hydrosols with a sharp distribution of particle sizes with a maximum in the region of 2.5–3.5 nm. It has been shown using the TEM, AFM, and DLS methods that the centrifugation of the “hydrosol/glycerol” system made it possible to get rid of aggregates and large diamond particles with sizes exceeding 5 nm. Moreover, the use of such a system allowed us to obtain DND hydrosols with a concentration of particles comparable to their concentration after simple centrifugation. The developed method, due to its simplicity and versatility, can be applied to all types of centrifuges and also scaled for industrial applications.

## Figures and Tables

**Table 1:** Percentage of DND particles with sizes less than 4 nm.

Method\Sample	DND-St	DND-SC	DND-MC
**TEM**	41.7%	71.8%	86.3%
**DLS**	55.4%	64.3%	93.3%
**AFM**	42.6%	48.4%	50.2%
